# Staphylococcal and Streptococcal Superantigens Trigger B7/CD28 Costimulatory Receptor Engagement to Hyperinduce Inflammatory Cytokines

**DOI:** 10.3389/fimmu.2019.00942

**Published:** 2019-04-30

**Authors:** Andrey Popugailo, Ziv Rotfogel, Emmanuelle Supper, Dalia Hillman, Raymond Kaempfer

**Affiliations:** Department of Biochemistry and Molecular Biology, Institute of Medical Research Israel-Canada, The Hebrew University-Hadassah Medical School, Jerusalem, Israel

**Keywords:** superantigens, inflammatory signaling, cytokine storm, costimulatory receptor engagement, CD28, B7-2, B7-1

## Abstract

Staphylococcal and streptococcal superantigens are virulence factors that cause toxic shock by hyperinducing inflammatory cytokines. Effective T-cell activation requires interaction between the principal costimulatory receptor CD28 and its two coligands, B7-1 (CD80) and B7-2 (CD86). To elicit an inflammatory cytokine storm, bacterial superantigens must bind directly into the homodimer interfaces of CD28 and B7-2. Recent evidence revealed that by engaging CD28 and B7-2 directly at their dimer interface, staphylococcal enterotoxin B (SEB) potently enhances intercellular synapse formation mediated by B7-2 and CD28, resulting in T-cell hyperactivation. Here, we addressed the question, whether diverse bacterial superantigens share the property of triggering B7-2/CD28 receptor engagement and if so, whether they are capable of enhancing also the interaction between B7-1 and CD28, which occurs with an order-of-magnitude higher affinity. To this end, we compared the ability of distinct staphylococcal and streptococcal superantigens to enhance intercellular B7-2/CD28 engagement. Each of these diverse superantigens promoted B7-2/CD28 engagement to a comparable extent. Moreover, they were capable of triggering the intercellular B7-1/CD28 interaction, analyzed by flow cytometry of co-cultured cell populations transfected separately to express human CD28 or B7-1. Streptococcal mitogenic exotoxin Z (SMEZ), the most potent superantigen known, was as sensitive as SEB, SEA and toxic shock syndrome toxin-1 (TSST-1) to inhibition of inflammatory cytokine induction by CD28 and B7-2 dimer interface mimetic peptides. Thus, superantigens act not only by mediating unconventional interaction between MHC-II molecule and T-cell receptor but especially, by strongly promoting engagement of CD28 by its B7-2 and B7-1 coligands, a critical immune checkpoint, forcing the principal costimulatory axis to signal excessively. Our results show that the diverse superantigens use a common mechanism to subvert the inflammatory response, strongly enhancing B7-1/CD28 and B7-2/CD28 costimulatory receptor engagement.

## Introduction

Bacterial superantigens are potent virulence factors secreted by *Staphylococcus aureus* and *Streptococcus pyogenes* that induce toxic shock by activating a cellular immune response, orders of magnitude greater than that elicited by regular antigens, leading to an 'inflammatory cytokine storm'. Classic work has shown that superantigens bind directly as intact proteins to most major histocompatibility class II (MHC-II) and T-cell receptor (TCR) molecules outside their antigen-binding domains, linking them while bypassing the restricted presentation of conventional antigens which typically activate less than 1% of T cells, thereby activating up to 20–30% of T cells (1–3). More recent work revealed that T-cell activation by superantigens requires, in addition, their direct binding to the principal costimulatory receptors, CD28 (4) and its coligand, B7-2 (CD86) (5). Together, these superantigen engagements result in a massive induction of inflammatory cytokines that mediate toxic shock, including interleukin-2, interferon-γ (IFN-γ) and tumor necrosis factor.

CD28 is a critical regulator of the immune response (6–8). Expressed constitutively on T cells, CD28 is a homodimer that interacts with its B7 coligands expressed on antigen-presenting cells, transducing the signal essential for T cell activation (7–10). Whereas B7-2 is expressed constitutively, CD28 coligand B7-1 (CD80) is induced gradually during the course of an immune response (10, 11); hence, the B7-2/CD28 interaction transmits the earliest signal induced by an antigen (12, 13).

Induction of inflammatory cytokine gene expression in human peripheral blood mononuclear cells (PBMC) by superantigen toxins depends on a 12 amino acid β-strand(8)/hinge/α-helix(4) toxin domain, remote from the MHC-II and TCR binding sites, that shows overall spatial conservation among diverse superantigens (14). A peptide mimetic of the β-strand(8)/hinge/α-helix(4) domain in the prominent superantigen, staphylococcal enterotoxin (SE) B, proved to be an effective antagonist not only of SEB but also of SEA, streptococcal pyrogenic exotoxin A (SPEA) and toxic shock syndrome toxin-1 (TSST-1), capable of attenuating the induction of inflammatory cytokines by these toxins in human peripheral blood mononuclear cells (PBMC) and of protecting mice from lethal challenge by each of these superantigen toxins (14–16). This finding led subsequently to the discoveries of CD28 (4) and its coligand B7-2 (5) as novel superantigen receptors to which the superantigen must bind in order to induce an inflammatory cytokine storm. Through their conserved β-strand(8)/hinge/α-helix(4) domain, essential for superantigen action (4, 14–16), superantigens engage CD28 directly at its homodimer interface (4). Moreover, using its β-strand(8)/hinge/α-helix(4) domain, the superantigen binds not only to the homodimer interface of CD28 but also to the crystallographic dimer interface of its coligand, B7-2 (5). This dual binding is critical for the induction of toxicity (4, 5). Inhibiting access of a superantigen to CD28, with short peptide mimetics of the β-strand(8)/hinge/α-helix(4) superantigen domain or of the CD28 homodimer interface, suffices to attenuate pro-inflammatory signaling by superantigens in human PBMC and protects mice from lethal superantigen challenge (4, 14, 17). Short peptide mimetics of the B7-2 dimer interface bind diverse superantigens, prevent binding of SEB to cell-surface B7-2 or CD28, inhibit superantigen-mediated induction of interleukin-2, IFN-γ and tumor necrosis factor in human PBMC, and are effective *in vivo*, protecting mice from lethal SEB challenge (5, 18).

A molecular mechanism for how this dual binding achieves signaling for T-cell hyperactivation was provided by study of SEB (5). Although in both CD28 and B7-2, the dimer interfaces (6, 19) are remote from the domains where these two costimulatory receptors engage one another, by binding into both dimer interfaces, SEB potently enhances the interaction between B7-2 and CD28 (5). Thus, SEB directly facilitates the interaction of B7-2 with CD28 to form the costimulatory axis (5).

Here, we asked whether the ability of SEB to trigger B7-2/CD28 receptor engagement represents a general property of the bacterial superantigen toxin family. Moreover, given that the B7-1 coligand binds CD28 with an order of magnitude higher affinity than does B7-2 (20), we asked whether superantigens might be capable of enhancing even the interaction between B7-1 and CD28.

## Materials and Methods

### Superantigens

SPEA was from Toxin Technology (Sarasota, FL). Chromosomal DNA isolated from *S. aureus* COL, from SEA- and TSST-1-producing strains of *S. aureus*, and from a SMEZ-producing strain of *Streptococcus pyogenes* was used to clone SEB, SEA, TSST-1 and SMEZ genes, respectively, into pHTT7K (21) and express them in *E. coli* as the mature proteins with an N-terminal His_6_-tag (4, 5). Inserts were verified by DNA sequencing. Total protein was loaded onto a His·Bind column (Novagen) and eluted stepwise with imidazole. Recombinant proteins recovered after dialysis were >98% pure on SDS-PAGE and >98% homogeneous as monomer upon analytical gel filtration through a 1 × 30 cm Superdex 75 column calibrated with molecular weight standards (GE Healthcare-Amersham Pharmacia) from which protein was eluted at a flow rate of 1 ml/min. Recombinant SEB was lethal to mice.

### CD28 and B7 Expression Vectors

Vectors expressing cell-surface CD28, CD28 fused C-terminally to GFP, cell-surface B7-2 and B7-2 or B7-2C fused C-terminally to Cherry have been described (4, 5). Vector expressing B7-1 was generated by cDNA synthesis of human CD80 (NM_005191.3) from total human PBMC RNA using Verso RT-PCR kit (ABgene). CD80 cDNA was generated using KOD polymerase (Novagen) with phosphorylated PCR primers 5′-GAC GTC GAC ATG GGC CAC ACA CGG AGG and 5′-CAC GCG GCC GCT TAT ACA GGG CGT ACA CTT TC CC. The PCR product was inserted into pEGFP-N3 DNA (Clontech) that had been digested with SalII and NotI and lacked the GFP region, using Fast-Link DNA Ligation Kit (Epicenter). Vector expressing B7-1 fused C-terminally to Cherry was generated from B7-1 cDNA vector template with phosphorylated PCR primers 5′-TAC TCG AGA TGG GCC ACA CAC GGA GG and 5′-GTC CGC GGT ACA GGG CGT ACA CTT TCC CT TC, deleting the *B7-1* termination codon. Upon digestion with XhoI and SacII, the PCR product was inserted into pmCherry-N1 DNA (Clontech).

### B7/CD28 Interaction

To assay the effect of superantigens on intercellular B7-2/CD28 synapse formation by flow cytometry, vectors expressing CD28/GFP and B7-2/Cherry fusion proteins were used that leave the extracellular ligand binding domains intact. HEK-293T cells, separately transfected with >75% efficiency using Turbofect Transfection Reagent (Thermo Scientific) and 6 μg of expression vector DNA per 5 ml of cells at a density of 10^5^/ml to express CD28/GFP (green) and B7-2/Cherry or B7-2C/Cherry (red), were co-incubated for 3 h at room temperature at a concentration of 10^5^ cells/ml each. Synapse formation between cell populations was analyzed by flow cytometry (Eclipse Flow Cytometry System, Sony), scoring the percentage of events positive for green and red using FlowJo vX.0.6 software. Contour plots were generated using FlowJo vX.0.6 software. Synapse formation between cells expressing CD28/GFP and B7-1/Cherry was assayed likewise.

### Induction of IFN-γ Expression

Human PBMC were separated on Ficoll Paque (Amersham), washed twice with 50 ml of RPMI 1640 medium, resuspended at 4 × 10^6^ cells/ml and cultured in this medium supplemented with 2% fetal calf serum, 2 mM glutamine, 10 mM MEM nonspecific amino acids, 100 mM Na-pyruvate, 10 mM Hepes pH 7.2, 50 μM 2-mercaptoethanol, 100 U/ml penicillin, 100 μg/ml streptomycin and 5 μg/ml nystatin. SMEZ was added to 10 ng/ml. Secreted IFN-γ was quantitated in triplicate with Quantikine ELISA kit (R&D Systems).

### Evolutionary Conservation of Superantigen Protein Sequences

Schematic models of protein structure were created in PyMol (www.pymol.org). To estimate the evolutionary conservation of amino acid positions in superantigen and superantigen-like protein molecules, based on phylogenetic relationships between homologous sequences, the ConSurf server was used [(22, 23); http://consurf.tau.ac.il/2016/]; amino acid sequences were analyzed using BLAST and multiple sequence alignment was performed using CLUSTALW.

## Results

### A β-Strand(8)/Hinge/α-Helix(4) Domain Is Conserved in Sequence and Structure Among Staphylococcal and Streptococcal Superantigens

**Figure 1:**
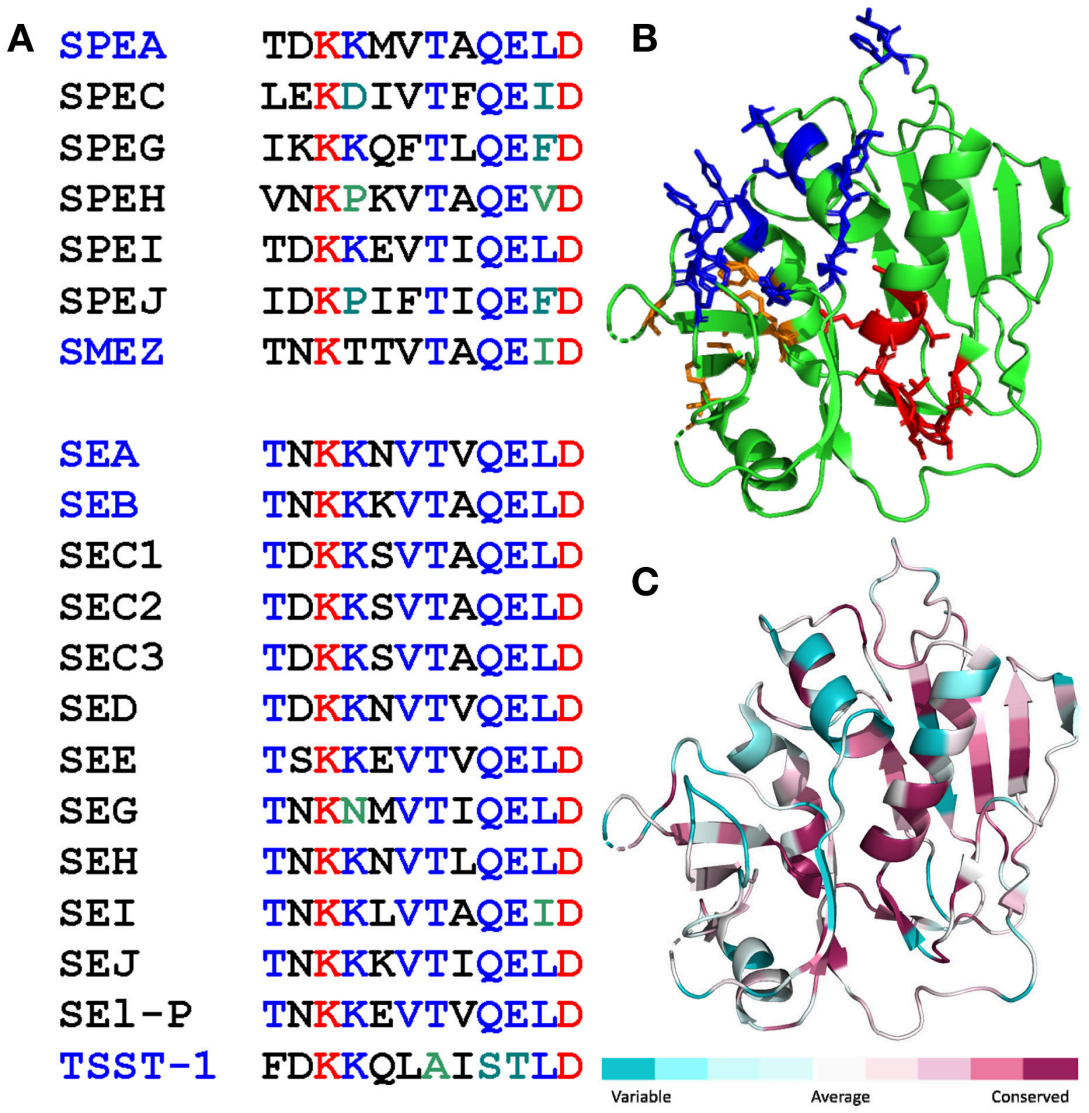
Conservation of the β-strand(8)/hinge/α-helix(4) domain in the bacterial superantigen family. **(A)** Amino acid sequences of the β-strand(8)/hinge/α-helix(4) domain (residues 145-156 in SEA) in representative streptococcal superantigens (top) and staphylococcal superantigens (bottom). Names of superantigens studied here are highlighted in blue. **(B,C)** The β-strand(8)/hinge/α-helix(4) domain shows high structural conservation among diverse superantigens (14). In cartoon structure of SEA [5fk9.pdb; (25)], the β-strand(8)/hinge/α-helix(4) domain is depicted in red, residues contacting the TCR (25) are in blue, and residues contacting the MHC-II (26) in orange **(B)**. Degree of amino acid sequence conservation among superantigens is mapped onto the SEA structure using Consurf **(C)**.

A dodecapeptide sequence within SEA, TNKKNVTVQELD, shows strong conservation among a broad range of staphylococcal and streptococcal superantigens (Figure 1A). We originally reported that in SEB, this sequence folds into a short β-strand(8)/hinge/α-helix(4) domain that is far removed from the domains that bind the classical superantigen receptors, MHC-II molecule and TCR, and indeed is located on the opposite side of the superantigen protein molecule (14). Overall folding of this domain, highlighted in Figure 1B for SEA, is conserved among SEA, SEB, SPEA and even TSST-1 which shares only 6% sequence homology with SEB (14). Indeed, when we compared the amino acid sequences of all the bacterial superantigens in a protein data base to that of SEA, the β-strand(8)/hinge/α-helix(4) domain was seated within the longest sequence showing high conservation (Figure 1C).

### Diverse Superantigens Promote B7-2/CD28 Costimulatory Receptor Engagement

Here, we examined whether the ability of SEB to promote B7-2/CD28 receptor engagement is shared by other bacterial superantigens. In order to study this specific interaction, we needed to devise a method that monitors the formation of the intercellular synapse between a cell expressing B7-2 on its surface and a cell that expresses CD28 on its surface. Notably, synapses formed between the antigen-presenting cell and the T cell involve not only the MHC-II/TCR interaction and the B7/CD28 interaction but also interaction between numerous additional costimulatory ligand pairs whose expression not only would confound measurement of intercellular synapse formation resulting specifically from B7-2/CD28 engagement but also could change as a result of exposure of the cells to a superantigen, affecting thereby synapse strength. To measure the B7-2/CD28 interaction in the absence of confounding ligand-receptor interactions, we used flow cytometry to quantitate formation of intercellular synapses mediated by CD28 and B7-2, each expressed in its native state on the membrane of transfected HEK293 cells (5). This allowed for selective monitoring of B7-2/CD28 synapse formation, in the absence of any MHC-II molecule or TCR.

**Figure 2:**
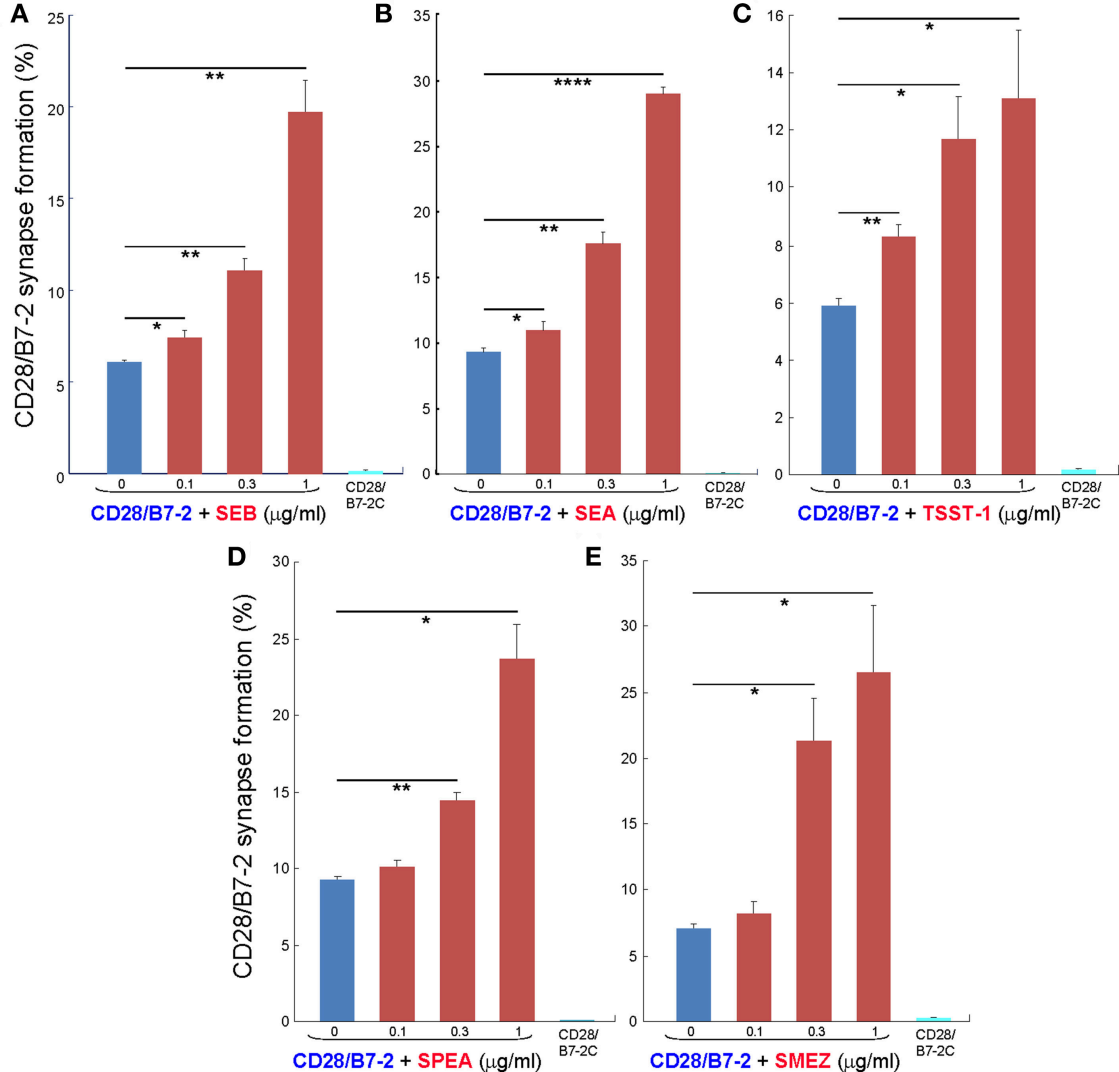
Staphylococcal and streptococcal superantigens trigger intercellular CD28/B7-2 synapse formation. **(A–E)** HEK293T cells transfected in triplicate to express CD28/GFP fusion protein (green label) were incubated with HEK293T cells transfected to express B7-2/Cherry fusion protein (red label), in absence (blue bars) or presence of the indicated superantigen at concentrations shown (red orange bars). As negative control served B7-2C/Cherry, which lacks the ability to bind CD28 (cyan bars). Intercellular CD28/B7-2-dependent synapse formation was scored using flow cytometry to quantitate per cent doubly labeled cells (error bars, SEM; *n* = 3). Comparisons were made using one-tailed unpaired Student's *t*-test; ^*^p < 0.05, ^**^p < 0.005, ^****^p < 0.0001.

**Figure 3:**
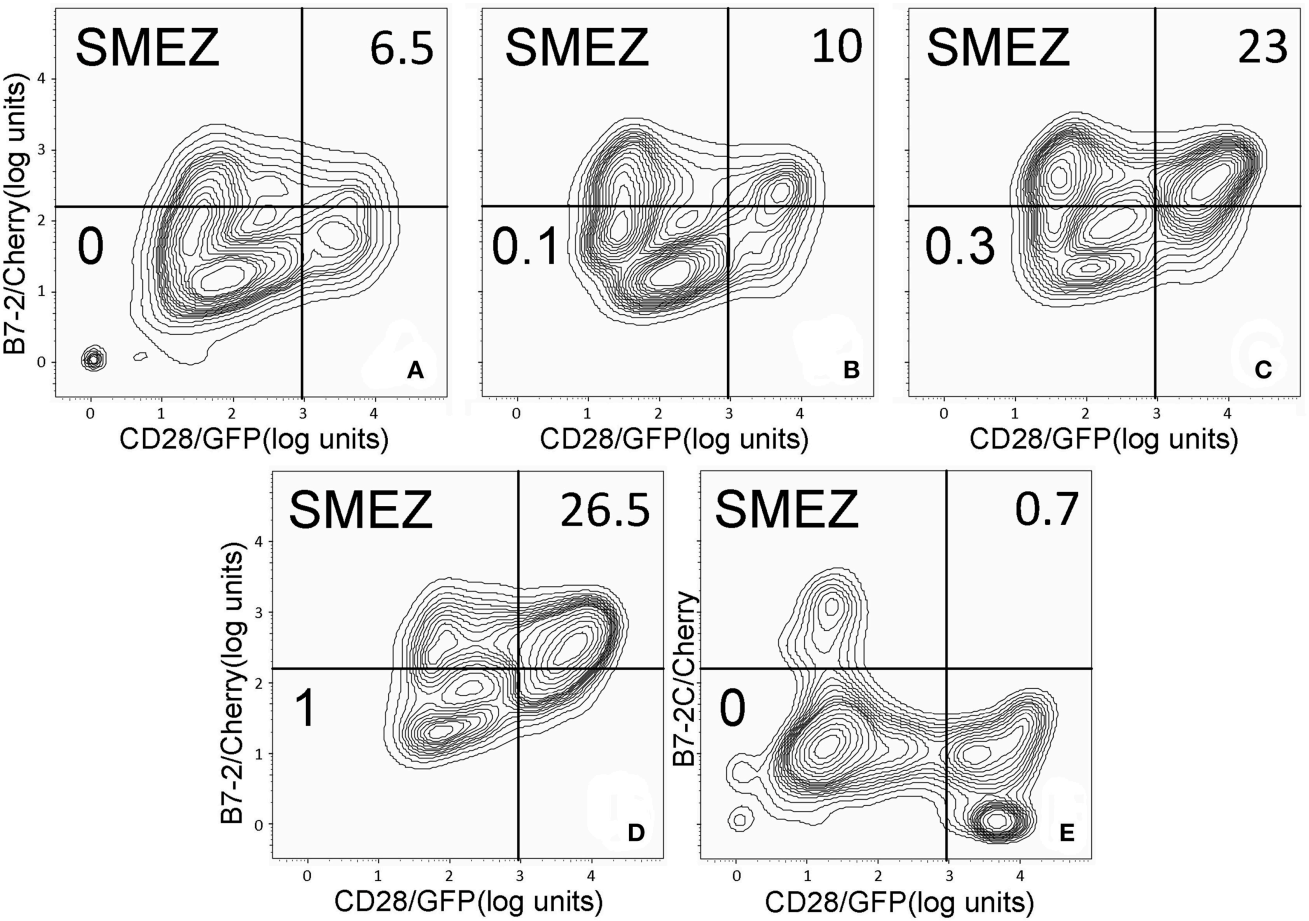
SMEZ triggers B7-2/CD28 synapse formation: contour plots. Contour plots are shown for a representative experiment in Figure 2E, upon incubation of cells expressing CD28/GFP with cells expressing B7-2/Cherry **(A–D)** or B7-2C/Cherry **(E)**. Incubation was done in the presence of the indicated concentrations of SMEZ from 0 to 1 μg/ml; per cent doubly labeled cells is shown in upper righthand corner of each panel.

As seen in Figures 2A–C, staphylococcal superantigen toxins SEA as well as TSST-1 showed an ability similar to that of SEB to enhance B7-2/CD28 synapse formation. Moreover, streptococcal superantigens SPEA and streptococcal mitogenic exotoxin Z (SMEZ) exhibited a comparable ability to promote B7-2/CD28 engagement (Figures 2D,E). Representative contour plots underlying the quantitative bar graphs of Figure 2 are illustrated for SMEZ in Figure 3. B7-, a splice variant of B7-2 that lost the ability to bind CD28 (27), failed to support significant synapse formation, demonstrating specificity of the interaction. Thus, each of these diverse superantigens shows a similar ability to promote B7-2/CD28 engagement.

**Figure 4:**
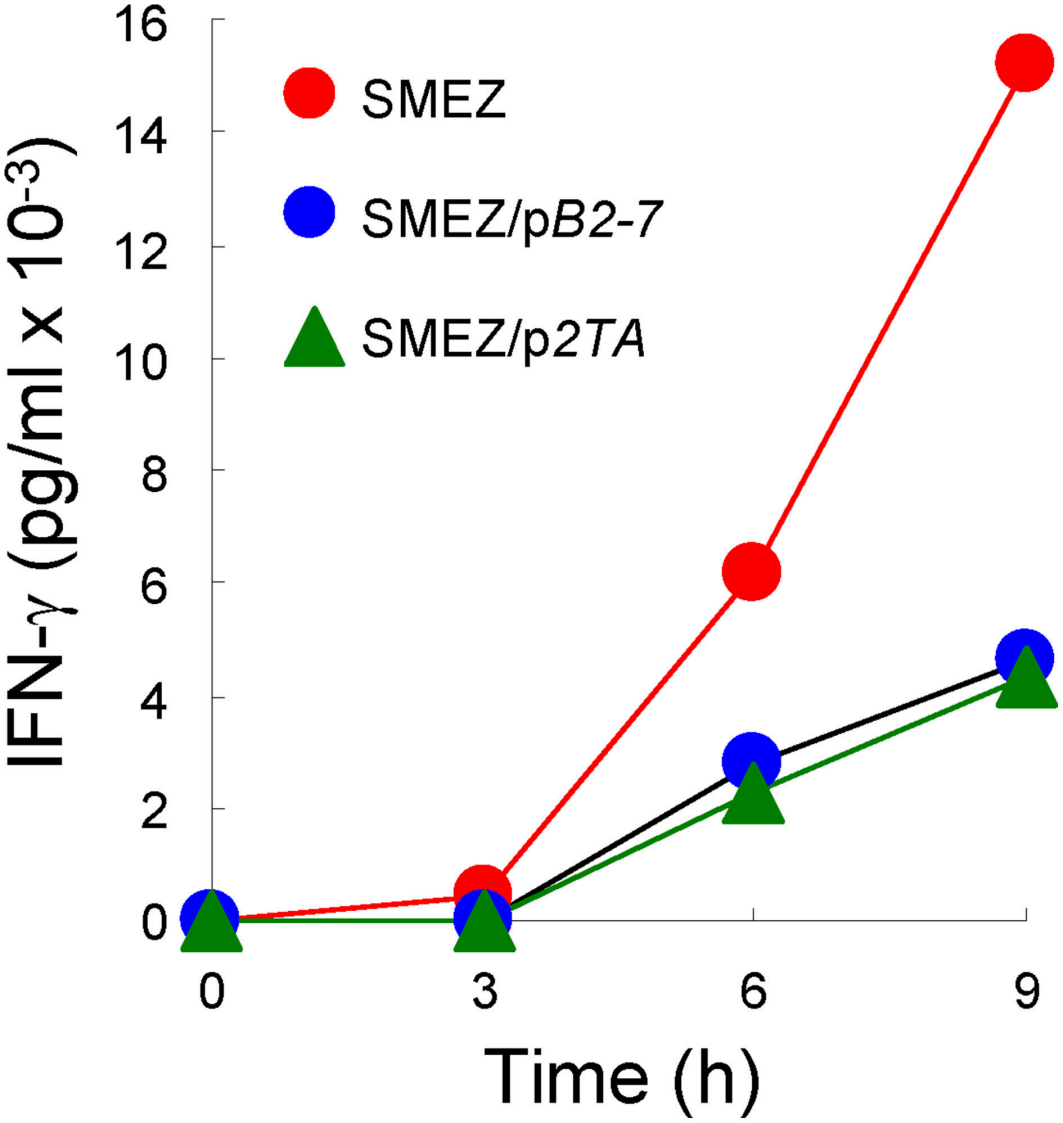
CD28 and B7-2 dimer interface mimetic peptides antagonize the ability of SMEZ to induce IFN-γ in human PBMC. PBMC were induced with SMEZ (10 ng/ml), in the absence or presence of 10 μg/ml B7-2 dimer interface mimetic peptide p*B2-7* or CD28 dimer interface mimetic peptide p*2TA*. Secreted IFN-γ is presented as means ± SEM; *n* = 3.

Independent evidence supporting a common mode of toxin action is furnished by the similar sensitivity of SEB, SEA and TSST-1 to inhibition by CD28- and B7-2-derived homodimer interface mimetic peptides (4, 5). As shown in Figure 4, despite its high toxicity, even SMEZ-induced expression of IFN-γ in human PBMC was attenuated to a closely comparable extent as for the other toxins by either CD28 dimer interface mimetic peptide p*2TA* (4) or B7-2 dimer interface mimetic peptide p*B2-7* (5).

### Diverse Superantigens Promote B7-1/CD28 Costimulatory Receptor Engagement

**Figure 5:**
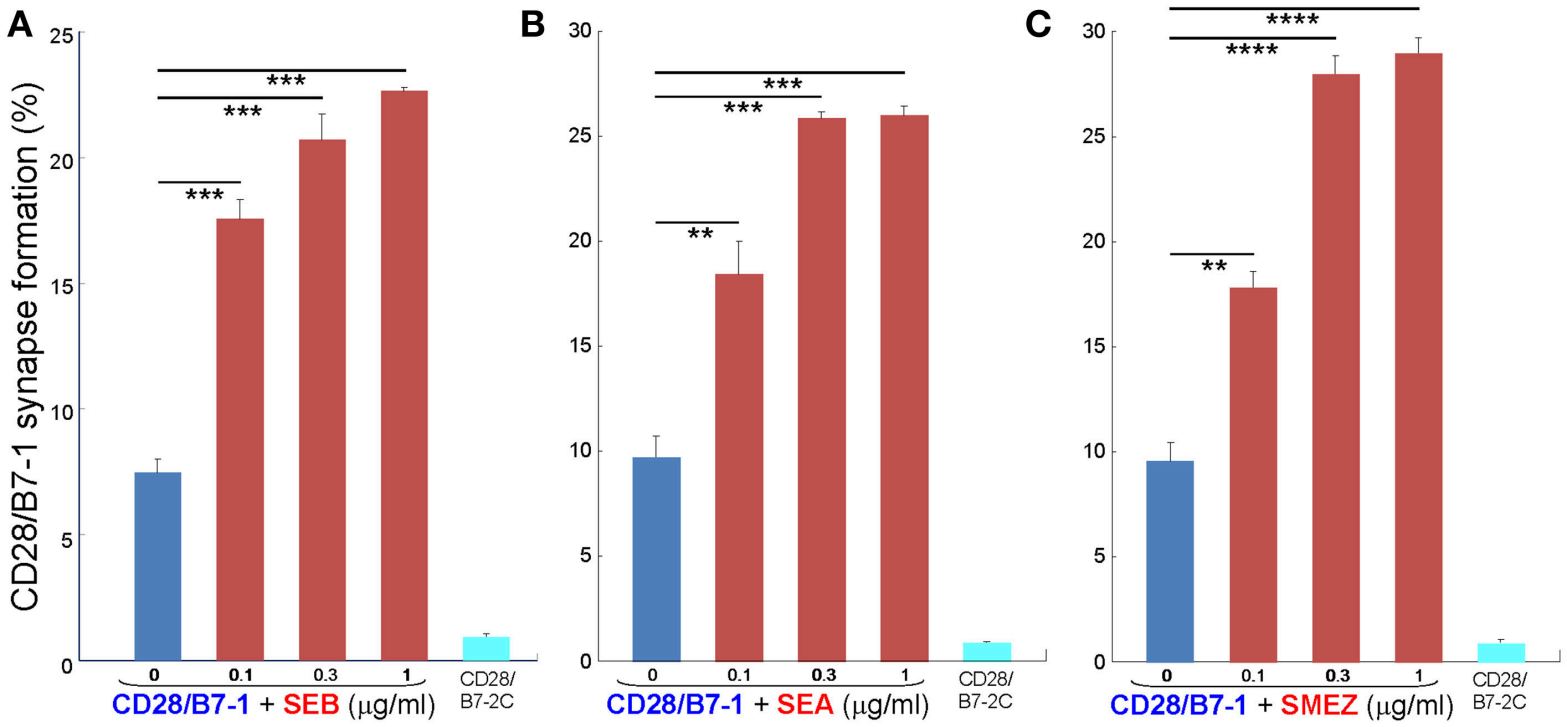
Staphylococcal and streptococcal superantigens trigger CD28/B7-1 synapse formation. **(A–C)** HEK293T cells transfected in triplicate to express CD28/GFP fusion protein (green label) were incubated with HEK293T cells transfected to express B7-1/Cherry fusion protein (red label), in absence (blue bars) or presence of the indicated superantigen at concentrations shown (red orange bars). As negative control served B7-2C/Cherry, which lacks the ability to bind CD28 (cyan bars). Intercellular CD28/B7-1-dependent synapse formation was scored using flow cytometry to quantitate per cent doubly labeled cells (error bars, SEM; *n* = 3). Comparisons were made using one-tailed unpaired Student's *t*-test; ^**^p < 0.005, ^***^p < 0.001, ^****^p < 0.0001.

**Figure 6:**
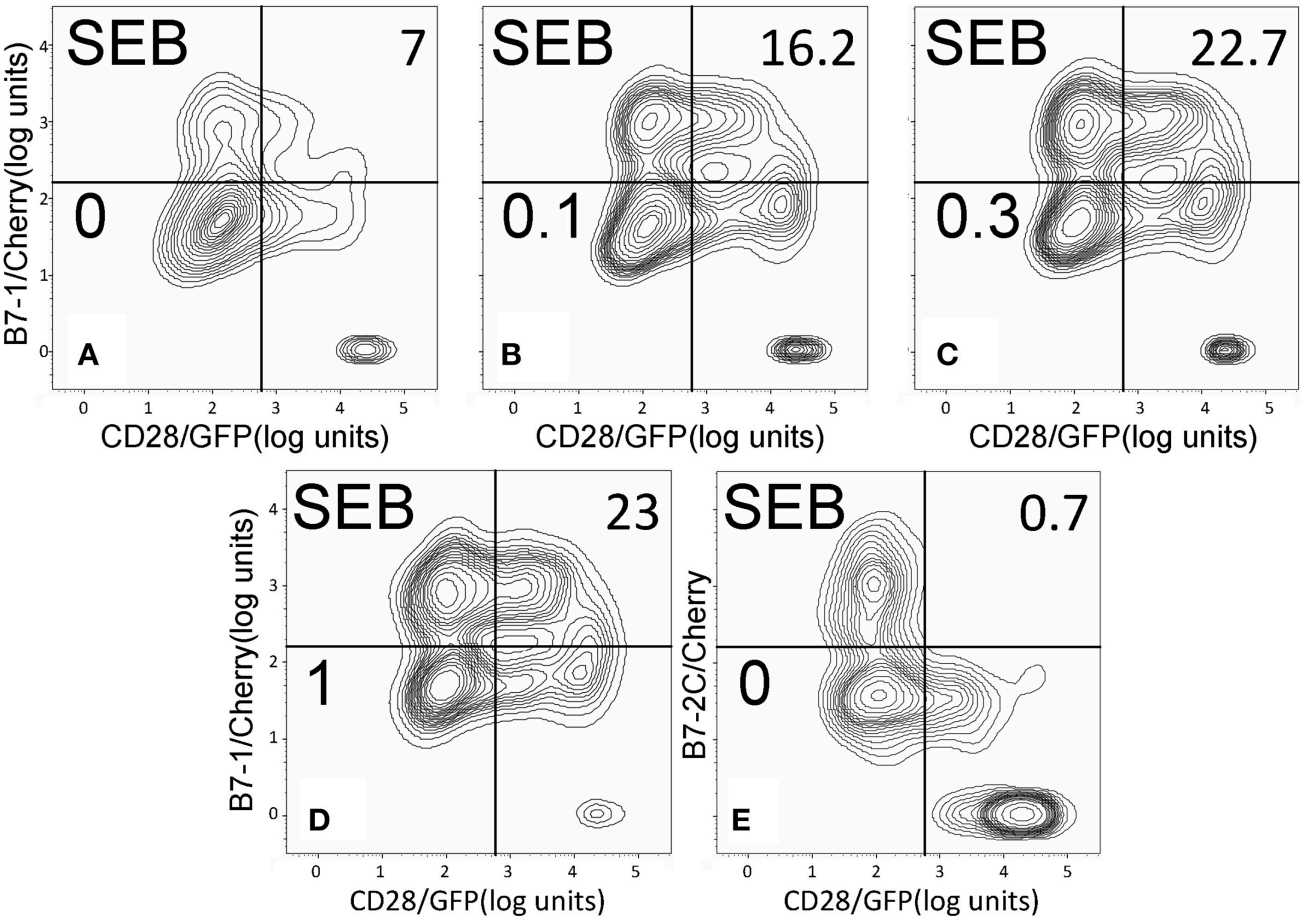
SEB triggers B7-1/CD28 synapse formation: contour plots. Contour plots are shown for a representative experiment in Figure 5A, upon incubation of cells expressing CD28/GFP with cells expressing B7-1/Cherry **(A–D)** or B7-2C/Cherry **(E)**. Incubation was done in the presence of the indicated concentrations of SEB from 0 to 1 μg/ml; per cent doubly labeled cells is shown in upper righthand corner of each panel.

Whereas the coligand B7-2 exhibits a low affinity for CD28 (K_D_, 20 μM), the second CD28 coligand, B7-1, binds CD28 far more strongly (K_D_, 5 μM) (20). We next examined whether superantigens can enhance also the B7-1/CD28 interaction. This is indeed the case. As seen in Figure 5, SEB, SEA as well as SMEZ each enhanced intercellular B7-1/CD28 synapse formation and did so to a similar extent. Corresponding contour plots are illustrated for SEB in Figure 6.

## Discussion

Our results show that the ability of diverse staphylococcal and streptococcal superantigens to bind directly to CD28 (4) and to B7-2 (5) is matched by a general ability to promote B7-2/CD28 engagement underlying formation of the primary costimulatory axis mandatory for T-cell activation. Indeed, these superantigens promote not only the between B7-2 and CD28 which occurs with low affinity but also the interaction between B7-1 and CD28 which occurs with far higher affinity. This property of the superantigen toxins can explain not only why they elicit an inflammatory cytokine storm resulting in lethality and toxic shock but also why homodimer interface mimetic peptides derived from CD28 or from B7-2 attenuate their ability to induce inflammatory cytokines (4, 5), illustrated here also for SMEZ. Diverse superantigens differ significantly in terms of their mode of interaction with the α- and β-chains of the MHC-II molecule: SEB, TSST-1 and SPEA bind only to the α-chain and SMEZ binds exclusively to the β-chain, whereas SEA engages both α- and β-chains (28, 29). Despite these pronounced differences in binding to MHC-II molecule, and a 40-fold difference in terms of toxicity between SEB and SMEZ (30), each of the superantigens we examined showed a very similar ability to promote B7-2/CD28 engagement. Moreover, this property extends to the ability of staphylococcal as well as streptococcal superantigens to enhance B7-1/CD28 engagement. Flow cytometry will not distinguish a synapse formed through a single intercellular B7/CD28 pair from one supported by multiple coreceptor pairs, yet each of the superantigens we tested showed a pronounced stimulatory effect on B7/CD28-mediated intercellular synapse formation, observed already at low toxin concentrations. We conclude that superantigens share the property of potently enhancing B7/CD28 costimulatory axis formation, critical for T-cell activation.

Thus, as initially demonstrated for SEB (5), bacterial superantigens uniquely facilitate not one but two molecular interactions that contribute to formation of the immunological synapse between antigen-presenting cell and T cell: interaction of MHC-II with TCR, acting as intermolecular bridge, and interaction of B7-2 as well as B7-1 with CD28 as shown here, forcing the principal costimulatory axis to signal excessively. As shown previously, SEB, SEA as well as TSST-1 each bind to CD28 at its homodimer interface (4) and SEB, TSST-1 as well as SMEZ each engage B7-2 at its crystallographic dimer interface (5), rendering their pro-inflammatory action sensitive to homodimer interface mimetic peptides (18). Data with a CD28 dimer interface mimetic peptide extend this property to SMEZ (Figure 4). Moreover, mice are protected from lethal challenge with SEB, SEA, SPEA as well as TSST-1 by a peptide mimetic of the conserved β-strand(8)/hinge/α-helix(4) superantigen domain (14–16) that superantigens use to bind directly to the dimer interfaces of CD28 (4) and B7-2 (5). The β-strand(8)/hinge/α-helix(4) superantigen domain does not interact with the MHC-II molecule (Figure 1B) (4, 14). Thus, it is not surprising that a peptide mimetic of this domain failed to block binding of SEB to MHC-II molecules (24) or, when applied in an excessively high dose far beyond the optimal range (14), to protect MHC-II transgenic mice from repeated challenges with SEB (24).

**Figure 7:**
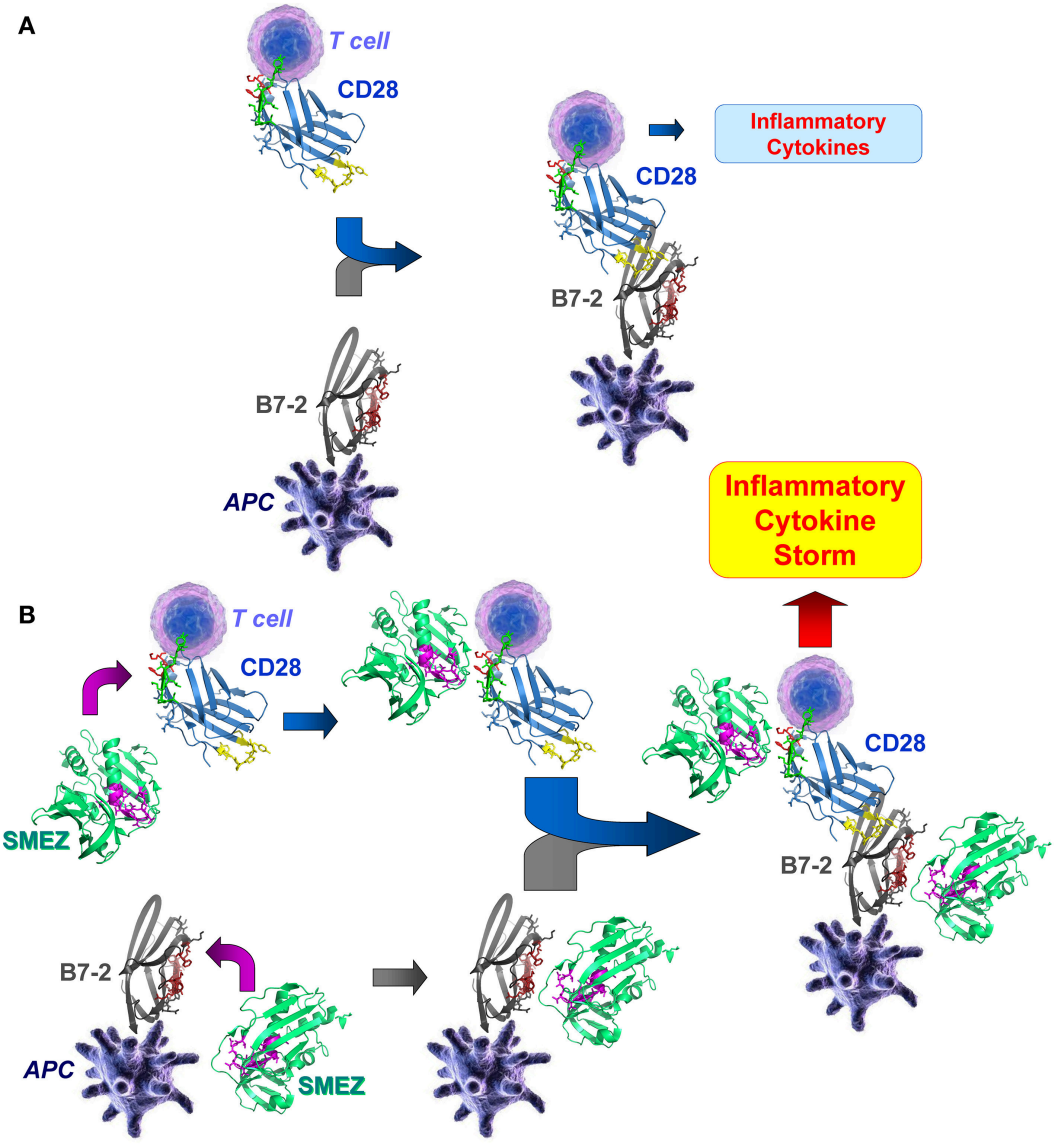
Superantigen binds directly into the homodimer interfaces of CD28 and B7-2, triggering B7-2/CD28 engagement that induces an inflammatory cytokine storm. Schematic diagram showing the interaction of a superantigen, SMEZ, with CD28 on the T cell and B7-2 on the antigen-presenting cell (APC). For clarity, the second monomer in the CD28 homodimer and engagement by SMEZ of TCR and MHC-II molecule were omitted. **(A)** Formation of the B7-2/CD28 costimulatory axis enables expression of inflammatory cytokines. **(B)** Two superantigen molecules bind, through their accessible β-strand(8)/hinge/α-helix(4) domain (magenta), B7-2 and CD28 at their homodimer interfaces within the extracellular domains. Binding of the superantigen potently enhances B7-2/CD28 engagement and inflammatory signaling.

The present results reveal a common mechanism utilized by a wide range of diverse superantigens, that is, to strongly enhance formation of the B7/CD28 costimulatory axis. This general mechanism of superantigen action is illustrated schematically in Figure 7 for the case of B7-2/CD28 engagement. Induction of inflammatory cytokines requires interaction between the antigen-presenting cell and the T cell, mediated by engagement of B7-2 by CD28 at binding sites well removed from their homodimer interfaces (Figure 7A). In the presence of a superantigen, illustrated here for SMEZ, direct binding of the superantigen to the CD28 and B7-2 dimer interfaces potentiates the B7-2/CD28 interaction, resulting in the induction of a harmful inflammatory cytokine storm (Figure 7B).

The folded homodimer interface of CD28 is highly composite, with regions both upstream and downstream from the compact B7 binding domain contributing dimer interface contacts (4, 19). The folded homodimer interface of B7-2 is also highly composite, as is its folded CD28 binding domain (5, 6). It is thus not surprising that allosteric effects can and do occur within these compact β-barrels. Mutating K118/K120 in the CD28 dimer interface (4, 19) enhanced the avidity of B7-1 binding (31). We have shown that engagement of the CD28 and B7 dimer interfaces by diverse superantigens strongly enhances intercellular synapse formation mediated by these costimulatory receptors.

## Author Contributions

AP, ZR, and RK designed the research. AP, ES, and DH conducted experiments. AP and RK analyzed the data and wrote the manuscript.

### Conflict of Interest Statement

The authors declare that the research was conducted in the absence of any commercial or financial relationships that could be construed as a potential conflict of interest.

## Abbreviations

SE: staphylococcal enterotoxin
SPEA: streptococcal pyrogenic exotoxin A
SMEZ: streptococcal mitogenic exotoxin Z
TSST-1: toxic shock syndrome toxin-1
MHC-II: major histocompatibility class II
TCR: T-cell receptor
IFN-γ: interferon-γ
PBMC: peripheral blood mononuclear cells
APC: antigen-presenting cell
K_D_: dissociation constant.
